# Cervical Endometriosis Mimicking Microinvasive Cervical Carcinoma: A Diagnostic Pitfall and Literature Review

**DOI:** 10.3390/life16071151

**Published:** 2026-07-12

**Authors:** Razvan Bobora, Ion Petre, Florina Buleu, Izabella Petre, Anca Bordianu, Marina Adriana Mercioni, Luciana Marc, Daian Ionel Popa, Flaviu Ionuț Faur, Tiberiu Buleu, Marius Furău, Laurentiu Cezar Tomescu

**Affiliations:** 1 Doctoral School, “Victor Babes” University of Medicine and Pharmacy Timisoara, Eftimie Murgu Square 2, 300041 Timisoara, Romania; boborarazvandaniel@gmail.com; 2 Department of Functional Sciences, Medical Informatics and Biostatistics Discipline, “Victor Babes” University of Medicine and Pharmacy Timisoara, Eftimie Murgu Square 2, 300041 Timisoara, Romania; 3 Cardiology Department, “Victor Babes” University of Medicine and Pharmacy Timisoara, 300041 Timisoara, Romania; 4 Department of Obstetrics and Gynecology, “Victor Babes” University of Medicine and Pharmacy Timisoara, Eftimie Murgu Square 2, 300041 Timisoara, Romania; petre.izabella@umft.ro; 5 Department of Plastic Surgery and Reconstructive Microsurgery, Bagdasar-Arseni, Emergency Hospital, 010825 Bucharest, Romania; anca.bordianu@gmail.com; 6 Faculty of General Medicine, “Victor Babes” University of Medicine and Pharmacy Timisoara, 300041 Timisoara, Romania; marina.mercioni@student.umft.ro (M.A.M.); tiberiu.buleu@umft.ro (T.B.); 7 Applied Electronics Department, Faculty of Electronics, Telecommunications and Information Technologies, Politehnica University Timisoara, 300223 Timisoara, Romania; 8 Center for Molecular Research in Nephrology and Vascular Disease, Faculty of Medicine, “Victor Babes” University of Medicine and Pharmacy Timisoara, Eftimie Murgu Square 2, 300041 Timisoara, Romania; marc.luciana@umft.ro; 9 Department of Internal Medicine II, Division of Nephrology, “Victor Babes” University of Medicine and Pharmacy Timisoara, 300041 Timisoara, Romania; 10 Research Center for Medical Communication, “Victor Babes” University of Medicine and Pharmacy Timisoara, Eftimie Murgu Square 2, 300041 Timisoara, Romania; daian-ionel.popa@umft.ro; 11 X Department of General Surgery, “Victor Babes” University of Medicine and Pharmacy Timisoara, 300041 Timisoara, Romania; flaviu.faur@umft.ro; 12 Emergency County Hospital “Pius Brinzeu”, 325100 Timisoara, Romania; 13 Romania-Medical Oncology Department, Emergency Clinical County Hospital of Arad, 310029 Arad, Romania; marius.furau@yahoo.com; 14 Faculty of General Medicine, “Vasile Goldiș” Western University of Arad, 310130 Arad, Romania; 15 Department of Obstetrics and Gynecology, “Ovidius” University of Constanta, 900527 Constanta, Romania; tomescu.cezar.laurentiu@gmail.com

**Keywords:** cervical cancer, cervical endometriosis, misdiagnosis, cervical cancer screening, gynecology, diagnostic pitfall

## Abstract

**Background:** Endometriosis of the cervix is a rare, non-cancerous disease that can look or behave like cancerous cervical tissue, causing challenges regarding diagnosis and management. Discrimination between benign and malignant lesions can be difficult when utilizing cytological, colposcopic, and histopathological data if the findings do not agree with each other. **Case Presentation:** We report the case of an asymptomatic 45-year-old woman who was initially diagnosed with FIGO stage IA1 microinvasive cervical carcinoma based on cytological, colposcopic, and conization findings. Further evaluation yielded persistent uncertainty in diagnostic imaging and, in addition, a concern for invasive cervical disease. Therefore, the patient underwent a total hysterectomy with bilateral salpingo-oophorectomy, and the result of the postoperative evaluation was cervical endometriosis without evidence of invasive malignancy. This example demonstrates the diagnostic challenges associated with rare benign cervical lesions, which may resemble cervical carcinoma, and emphasizes the high-risk for overtreatment due to the vagueness of these types of cases. **Conclusions:** When there is a discrepancy among clinical findings, cytology, and histopathology in suspicious cervical lesions, cervical endometriosis must be considered in the differential diagnosis. To increase accuracy in diagnosis and minimize the possibility of unnecessary radical treatment, careful histopathologic reevaluation and multidisciplinary assessment are necessary.

## 1. Introduction

A benign condition of the female reproductive system characterized by the presence of abnormal endometrial glands or stromal tissue (the supporting connective tissue) outside of the uterus is called endometriosis. While pelvic organs are usually where endometriosis occurs, involvement of the cervix is uncommon and is not often identified in current medical practice [1].

Cervical endometriosis has clinical significance in that it can produce signs and symptoms similar to those of malignant (or premalignant) cervical lesions. Because cervical endometriosis, high-grade squamous intraepithelial lesions and invasive cervical neoplasia share several morphological similarities, the interpretations from cervical cytology, colposcopy, and even histopathological examination of cervical specimens may be deceptively similar [2,3].

Due to asymptomatic patients and reliance on histopathological confirmation, the true prevalence of endometriosis in the cervix is still unknown. Diagnosis is established via biopsy or surgical excision, not by clinical examination. Possible mechanisms of cervical endometriosis include implantation after cervical injury, such as from biopsies, electrosurgery, or childbirth, and metaplastic transformation of the cervical stroma or epithelium [4,5].

Cervical endometriosis remains a diagnostic pitfall for clinicians due to its rarity, minimal contemporary data, and limited awareness among healthcare providers. To illustrate this point, a case of cervical endometriosis misdiagnosed as cervical carcinoma will be provided along with a narrative review of previously published cases to facilitate the understanding of diagnostic error and offer clinical recommendations to minimize overtreatment. Artificial intelligence (AI) was only discussed in relation to possible future applications [6].

Epidemiological and molecular findings indicate that endometriosis possesses malignancy potentials that play a role in ovarian cancers [7]. The occurrence of endometriosis was observed in 39.2% of cases of clear cell ovarian cancer and 21.2% of endometrioid ovarian cancer cases, in contrast to 3.3% and 3% in severe and mucinous subtypes, respectively [8].

The ongoing enhancement of screening methodologies has led to an increased rate of detection for both cervical cancer and cervical endometriosis; nevertheless, the majority of fatalities occur in low- and middle-income nations [9,10,11,12]. Although advances have been made in effective screening initiatives, their implementation and sustainability are often hindered by inadequate health infrastructure. Furthermore, manual screening does not consistently achieve 100% accuracy, which can result in some lesions related to both conditions being definitively diagnosed only postoperatively when histopathologic evaluation is conducted, consequently delaying the diagnosis of cervical endometriosis [13]. Therefore, the primary challenge for the timely identification of both cervical cancer and cervical endometriosis lies in the development of a more precise and cost-effective approach, such as artificial intelligence (AI)-based medical diagnostic applications tailored for screening and diagnosing cervical pathologies. However, artificial intelligence has emerging applications in cervical cytology and digital pathology, with its role in detecting rare benign cervical lesions being limited.

This case report aims to highlight the diagnostic complexity of cervical endometriosis mimicking cervical malignancy and emphasizes the importance of careful histopathological reassessment and multidisciplinary evaluation in diagnostically ambiguous cervical lesions.

## 2. Case Report and Literature Review

### 2.1. Case Presentation

**Figure 1:**
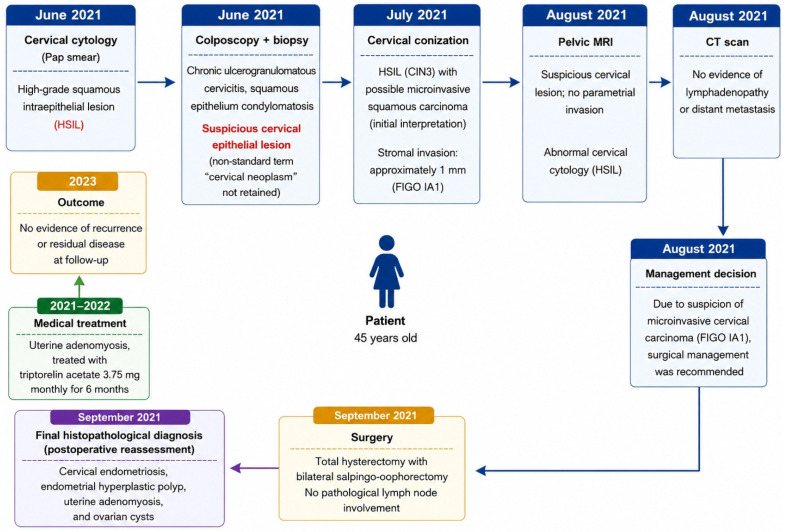
Chronological timeline of the diagnostic evaluation, therapeutic interventions, and final histopathological reassessment. Abbreviations: HSIL, high-grade squamous intraepithelial lesion; CIN3, cervical intraepithelial neoplasia grade 3; MRI, magnetic resonance imaging; CT, computed tomography; FIGO, International Federation of Gynecology and Obstetrics.

A 45-year-old woman without any clinical symptoms presented to our clinic in June 2021 with an anatomopathological result after a routine gynecological examination was performed at a private center, where prior PAP testing, colposcopy, and conization were conducted, with a high-grade squamous intraepithelial lesion (HSIL) revealed by the Pap test. The most critical stages in the patient’s evolution are represented in Figure 1.

Routine blood, liver, and kidney tests; coagulation tests; and electrolyte levels were normal. Serum β-HCG testing was negative. Transvaginal ultrasound was not extensively documented in this case, which is a limitation, as specialized ultrasound evaluation may improve lesion characterization.

**Figure 2:**
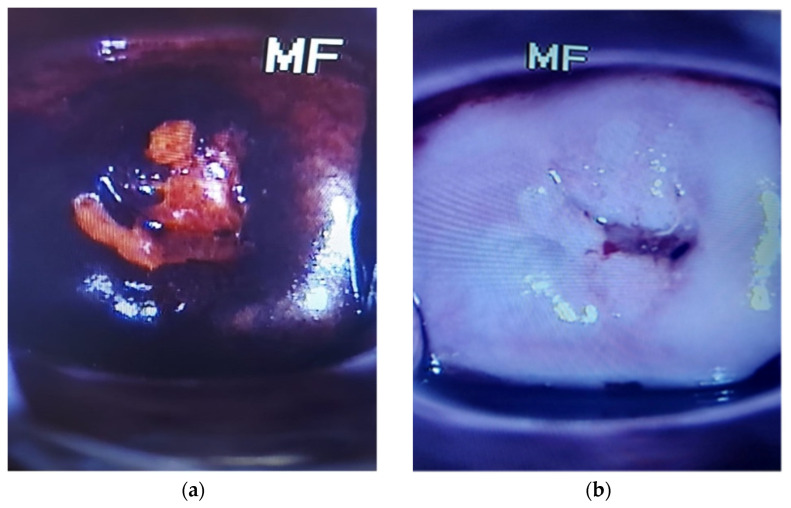
Colposcopy aspects of the post-cervix examination with the solution (**a**) Lugol and (**b**) acetic acid.

Subsequent investigation through colposcopy and biopsy of the tumor confirmed the presence of cervical cancer. Colposcopy images are presented in Figure 2. These findings, including acetowhite changes and iodine-negative areas, are commonly associated with high-grade intraepithelial lesions, which contributed to the initial misinterpretation.

Conization was performed, resulting in a histopathological examination revealing a cervical fragment. The conization cone, measuring 12 mm in height, was excised along the flange, and serrated sections were taken for complete processing. The resection margins were marked with a green tusk. The diagnosis indicated chronic ulcer granulomatous cervicitis, condylomatous squamous epithelium showing severe dysplasia (CIN gr. III), and carcinoma in situ with involvement of endocervical glands (high-grade intraepithelial squamous lesion). A limited area of microinvasion, measuring 1 mm in surface area and 1 mm in depth, was also identified. Microinvasive carcinoma was diagnosed only microscopically, with no lymphovascular involvement and tangential resection edges along the endocervical dysplastic epithelium. The presence of glandular involvement and focal microinvasion further reinforced the suspicion of malignancy, contributing to the diagnostic error.

Colposcopic examination demonstrated a circumscribed lesion with iodine-negative areas using Lugol staining and changes in acetowhite status following acetic acid application, indicating that this is consistent with a high-grade intraepithelial lesion.

In August 2021, the patient underwent a computerized tomography (CT) scan of the abdomen and pelvis. The examination revealed a homogeneous uterine structure with iodine uptake. There was a distinct demarcation from neighboring structures, and no periaortic, celiac, mesenteric, or adenopathies were observed in the renal or inguinal–pelvic region. Furthermore, all other organs scanned by CT appeared normal.

Subsequently, pelvic magnetic resonance imaging (MRI) was performed, indicating a diagnosis of a uterine tumor. The uterus was found in anteroflexion, with dimensions measuring up to 73.5/46.4 mm, displaying a linear endometrium and a diffuse hyper-trophied junctional area measuring up to 15.2 mm. Additionally, mild native and post-contrast endometrial thickening was observed, along with small uterine fibroids, moderate engorgement of para-uterine venous plexuses, and fluid accumulation at the bottom of the Douglas pouch. The bladder showed normal volume, without lesions on the wall or within the cavity. The rectosigmoid was normal, with no obvious intrapelvic lymphadenopathy or secondary pathological changes in the pelvic bones. Lymphadenectomy was performed during the surgical procedure.

A cervical biopsy was conducted after a high-grade squamous intraepithelial lesion (HSIL) was detected following a cervical smear. The first histological evaluation gave an impression of an abnormal cervical epithelium; however, the authors have subsequently changed the descriptive terminology to ‘abnormal cervical epithelial growth’ because ‘cervical cancer’ does not meet the guidelines of the World Health Organization (WHO) and the College of American Pathologists (CAP) classification systems for histopathological diagnoses. Following conization and a second review by an independent pathologist, the conization specimen was initially diagnosed as a possible invasive squamous carcinoma (FIGO Stage IA1) because deep malignant invasion into the stroma was not apparent based on the previous histopathological findings. The subsequent (i.e., final) histopathological and surgical findings did not support the presence of malignancy but indicated the presence of cervical endometriosis.

The discharge diagnosis included carcinoma in situ of the cervix without further specification. A secondary diagnosis of uterine leiomyoma was also noted without specific details. Functional disorders of neutrophil polynucleate were also observed during biological reevaluation. This functional disorder is known to be a risk factor and was identified as a uterine tumor with an unpredictable and unknown evolution [14]. To resolve this uncertainty, an oncological consultation was performed during hospitalization, which yielded the diagnosis of cervical cancer biopsied by conization. We recommend the patient undergo a total hysterectomy with bilateral salpingo-oophorectomy and postoperative oncologic reevaluation and treatment. The woman refused, at that moment, the intervention and was discharged under good general conditions, that is, afebrile with normal appetite, regular bowel transit, physiological micturition, a soft and mobile abdomen, and absence of vaginal pain or bleeding. She was advised to return for surgery.

In September 2021, she returned, and the surgery was performed, involving a total hysterectomy with bilateral salpingo-oophorectomy, with favorable postoperative recovery.

Subsequently, the histopathological examination results of the paraffin-embedded tissue were analyzed. Histopathological examination using complete Hematoxylin–Eosin (HE) staining was also performed. Regarding the cervical region, the following observations were made: a nonspecific subacute uterine cervix with areas of ulceration of the endocervical mucosa, mature squamous metaplasia, dilated cystic glands and foci of tubo-endometriotic metaplasia. In the endocervical wall, interstitial fibrosis, hyperemic vessels, hematic sweating, large-caliber vessels with thickened fibrous walls and dystrophic calcifications were observed; in addition, multifocal lesions of cervical endometriosis were found, predominantly in the superficial exocervical chorion. The squamous epithelium showed discrete inflammation and reactive changes without apparent tumor aspects. The uterine corpus showed a histologically benign endometrium in the proliferative phase, a hyperplastic endometrial polyp, and florid adenomyosis lesions at the isthmic level. Serous uterine tissue showed no significant histological changes. Furthermore, the conclusions drawn from this examination were that cervical endometriosis was associated with nonspecific subacute cervicitis lesions, a hyperplastic endometrial polyp, and uterine adenomyosis, and bilateral functional ovarian cysts were also noted.

Postoperatively, the patient received treatment comprising a monthly dose of 3.75 mg of Diphereline for 6 months (2021–2022). In 2023, the control pelvic and abdominal MRI revealed total post-hysterectomy with bilateral salpingo-oophorectomy without detectable adenopathies. The application of a GnRH agonist (Diphereline) for postoperative treatment was begun; however, it may be prudent to seek alternative means of treatment, such as dienogest, since they may offer greater tolerance for continued long-term use.

In this instance, AI was not utilized in any part of the diagnostic process and thus remains in a theoretical capacity, and it is explained from a speculative point of view only.

This case is an example of a complete change in diagnosis over time; the patient’s original diagnosis of a high-grade squamous intraepithelial lesion (HSIL) and microinvasive cervical cancer was changed to benign cervical endometriosis after undergoing a second review of histologic specimens.

#### Ethical Considerations

The clinical case was conducted in accordance with the principles outlined in the Declaration of Helsinki. Written informed consent was obtained from the patient for the use of her anonymized clinical data and histopathological images for research and publication purposes. Institutional review board (IRB) approval was not required for this single-case report, in compliance with local ethical guidelines.

### 2.2. Literature Review

A narrative literature review was conducted to identify published case reports and series describing patients initially diagnosed with cervical cancer or premalignant lesions who were ultimately found to have cervical endometriosis. The following electronic databases were searched: PubMed, Scopus, Google Scholar, and Web of Science. A thorough search was conducted using combinations of the following terms: “cervical endometriosis”, “cervical cancer misdiagnosis”, “ectopic endometrial tissue”, “gynecologic malignancy”, “HSIL differential diagnosis”, and “artificial intelligence in gynecology”. All duplicates were eliminated, and references from the selected case reports were manually examined for any qualifying studies. Furthermore, the bibliographies of the articles and review papers obtained were cross-referenced to ensure that all relevant case reports fitting our search criteria were included. A manual search of conference abstracts, posters, and other databases for case reports was performed to enhance our review. We included in our final sample (1) articles published in English between 2000 and 2024, (2) case reports or case series involving cervical endometriosis with or without coexisting malignancy, and (3) studies that discussed diagnostic methods or misdiagnoses. Furthermore, the exclusion criteria also included animal studies, non-cervical endometriosis cases, and review articles without original patient data. Titles and abstracts were screened by two independent reviewers. The full texts of relevant articles were reviewed for clinical presentation, diagnostic techniques, differential diagnoses, and treatment. So, a total of 124 articles were identified, with 32 duplicate articles removed, 67 excluded after title/abstract review, and 17 evaluated based on their full text, of which 9 did not meet the inclusion criteria: misdiagnosed cervical endometriosis as cervical cancer.

Eight articles met the criteria for inclusion in this review. Notably, all articles were case reports discussing cervical endometriosis that was initially diagnosed as serous papillary carcinoma of the cervix, as summarized in Table 1.

Our summary of case reports of cervical endometriosis initially misdiagnosed as gynecological cancer from the literature showed that cervical endometriosis is a rare and often underrecognized condition whose presentation ranges from dramatic to entirely silent. Most patients present with troublesome symptoms, including persistent intermenstrual bleeding, postcoital spotting, pelvic pain, or infertility [4,15,16,17,18,19,20,21,22], although a small proportion is diagnosed incidentally during evaluation for unrelated concerns [16,19]. Imaging, when available, may misleadingly suggest uterine fibroids, benign cysts, or vascular cervical masses [15,18,20,22], yet in more than half of the reported cases, no imaging findings are documented [4,16,17,19,21]. The clinical picture is further complicated by its ability to mimic cervical cancer, polyps, dysplasia, or other benign tumors, often delaying the correct diagnosis of cervical endometriosis [4,15,16,17,18,19,20,21]. Definitive identification relies on histopathological confirmation via biopsy, histology, or laparoscopy [4,15,16,17,18,19,20,21].

Management most commonly involves surgical excision [4,18,19,20,21,22], which is sometimes supplemented by hormonal agents such as dienogest [15,22]. While follow-up data are limited, available reports suggest that treatment is generally curative, with most patients experiencing symptom resolution [19,22]. A recent case report from China in 2024 [22] illustrates the following: a woman with recurrent vaginal bleeding was found to have a cystic cervical lesion using MRI. Surgical removal followed by hormonal therapy resulted in eight months of complete, symptom-free recovery, underscoring the potential for excellent outcomes when the condition is recognized and treated appropriately.

## 3. Discussion

Endometriosis in the cervix is a rare form of endometriosis and a major diagnostic difficulty, as it can appear in the clinical, cytological, and histopathological assessments to be a benign or malignant cervical lesion [10,16,17]. The case described in this report demonstrates the challenges in differentiating between benign endometriosis of the cervix and microinvasive squamous carcinoma of the cervix when the results of cytology, colposcopy, imaging, and histopathology are all inconsistent or differing. Specifically, the initial cervical cytology case from this patient showed a high-grade squamous intraepithelial lesion, while the conization of the cervix suggested that there may be a very early microinvasive squamous carcinoma at the IA1 stage (based on FIGO classification) that has focal stromal invasion. These results led to a surgical radical technique being performed on this patient, but at the time of the histopathological assessment of the resected specimen, there was no evidence of an invasive malignancy; therefore, the final diagnosis was endometriosis of the cervix without evidence of malignancy. Due to the lack of a separate review of the original conization specimen, it cannot be ruled out that an extremely limited area of microinvasive squamous carcinoma was successfully removed during the conization procedure. Therefore, the case discussed should be considered as having a complex pathological lesion in which cervical endometriosis was present at the same time that microinvasive cervical carcinoma was clinically indicated based on the initial pathology findings.

Numerous studies indicate that cervical endometriosis can mimic the characteristics of cervical neoplasia due to overlapping morphological and cytological similarities lesion [10,16,17]. For example, some colposcopic findings (i.e., vascular abnormalities, iodine-negative areas from applied Lugol’s stain, or irregular cervical lesions) often exhibit highly similar characteristics to the histopathological findings for squamous intraepithelial lesions or invasive cervical carcinoma. Additionally, interpretation of the histopathological findings can be particularly challenging with inflammatory changes, glandular involvement, reactive atypia, or any areas possibly indicating stromal invasion [23]. The present example supports the view that the presence of chronic ulcerogranulomatous cervicitis, CIN III, glandular involvement and an area deemed to show possible microinvasion in this case significantly added to the initial suspicion of malignancy.

In this manuscript, the literature review found only a small number of published cases documenting that cervical endometriosis had been misdiagnosed as cervical cancer or a precancerous lesion [4,15,16,17,18,19,20,21,22]. Most women had presented with abnormal bleeding, pelvic pain, and infertility or had developed visible lesions on their cervix that were noted at routine gynecological examinations, and there were frequent ambiguous x-ray findings, with some being suggestive of cancer [18,20,22]. Similar to our patient, many of the cases previously reported underwent substantial diagnostic and surgical treatment before it was histologically established that they had cervical endometriosis [18,19,20]. Thus, the findings within those reports confirm that it is a rare condition and that there continues to be difficulty in differentiating cervical endometriosis from malignancy of the cervix.

Our case provides important insights concerning possible overtreatment in cases of diagnostic ambiguity of cervical lesions. While the surgical decision was made based on an initial interpretation that the cervical lesion was an invasive carcinoma (FIGO IA1), the final diagnosis revealed it to be benign. Published cases have provided similar examples of this phenomenon, especially in cases of uncommon gynecologic diseases where there are similarities in their pathological characteristics [17,18,20]. Therefore, clinicians and pathologists should remain vigilant as rare benign cervical lesions (e.g., cervical endometriosis) can be difficult to differentiate from cervical malignancy, and continued diagnostic ambiguity could lead to overly aggressive management.

From the literature search, most of the women present with persistent intermenstrual bleeding, postcoital spotting, pelvic pain, or infertility [4,15,16,17,18,19,20,21,22], while others remain asymptomatic until diagnosis is made during routine or unrelated examinations [16,17,19]. Imaging findings, when available, are often nonspecific. Reported appearances include fibroids, benign cysts, or vascular cervical lesion [15,18,20,22], although in many cases, imaging is either not performed or not reported [4,16,17,19,21]. This lack of specificity contributes to frequent misdiagnoses, with cervical cancer, polyps, dysplasia, and other benign tumors often suspected first [4,15,16,17,18,19,20,21,22]. As a result, a definitive diagnosis relies on histopathological confirmation via biopsy, histology, or laparoscopy [4,15,16,17,18,19,20,21,22].

Management is primarily surgical, with lesion excision the most widely reported approach [4,15,18,19,22]. Hormonal therapy, such as dienogest, may be used as an adjunct in selected cases [15,22]. While the published follow-up data remain limited, available reports suggest favorable outcomes, with most patients achieving complete symptom resolution [22]. Contemporary NCCN and ESGO/ESTRO/ESP guidelines state that in certain circumstances, conization may be adequate for the treatment of selected FIGO IA1 microinvasive squamous carcinoma cases (diagnosed with negative margins and without lymphovascular space invasion). In our case, the surgical management decision was supported due to continued diagnostic uncertainty and a concern for the presence of invasive cervical carcinoma.

Unfortunately, Romania ranks first in Europe for the number of cervical cancer cases, with treatment costs being extremely high [24,25]. This is largely because most cases are detected at advanced stages rather than early on [25], a consequence of inadequate screening programs and low HPV vaccination coverage [26].

Even with several new developments in the field (cervical cytology, colposcopy, imaging, and histopathological techniques), it is still difficult to accurately diagnose uncommon benign cervical lesions. There are emerging uses of artificial intelligence in both cervical cytology and digital pathology, but very little use of AI for the assistance of the diagnosis of rare benign cervical lesions exists. Multidisciplinary clinical assessment, as well as a careful reassessment of histopathology, is critical to establish diagnostic precision in complex cases at this time [27].

This report has several limitations. It is a single case from which generalizability of findings cannot be made; imaging and ultrasound data had certain details that were incomplete because the patient’s initial investigations were conducted in a facility that did not provide them with all of the information; and retrospective reinterpretation of histopathology will potentially create interpretative bias. However, the educational aspect of the case demonstrates the diagnostic complexity often present with regard to diagnosing cervical endometriosis and highlights the critical need for careful reevaluations of pathologic specimens so that women are not subjected to unnecessary radical treatment.

Cervical endometriosis is an uncommon non-malignant condition that can closely resemble malignant lesions of the cervix across all examination modalities, including cytology, colposcopy, imaging, and histopathology. Discordance between clinical observations and pathological findings of the cervix should raise clinical suspicion for cervical endometriosis in differential diagnoses. A thorough reassessment of histopathological data and a multidisciplinary approach will assist in improving diagnostic accuracy and reducing the rate of unnecessary treatment.

## 4. Conclusions

Cervical endometriosis is a rare but clinically significant condition that can mimic cervical cancer and result in a misdiagnosis. Our case, along with the literature review, highlights the importance of maintaining a high index of suspicion, particularly when paraclinical investigations yield conflicting results. Histopathological confirmation remains the gold standard for diagnosis. The review also shows that most cases are either misdiagnosed or identified incidentally, underscoring the need for heightened clinical awareness. Recognizing this diagnostic pitfall can help clinicians avoid unnecessary radical interventions and ensure timely, appropriate management. This case highlights the diagnostic complexity of cervical endometriosis presenting alongside pathological findings suspicious for microinvasive cervical carcinoma and emphasizes the importance of careful histopathological reassessment and multidisciplinary evaluation in ambiguous cervical lesions. While emerging technologies such as artificial intelligence may support future diagnostic processes, clinical judgment remains essential.

## Figures and Tables

**Table 1:** Summary of case reports of cervical endometriosis initially misdiagnosed as gynecological cancer from the literature.

No.	Author(s)/Year	Country	Age	Presenting Symptoms	Imaging Findings	Initial Diagnosis	Final Diagnosis (Method)	Treatment	Follow-Up/Outcome	Ref.
1	Atalay Mert et al., 2023	Turkey	38	Pelvic pain, menstrual irregularity; adenomyosis	MRI: focal adenomyosis and small leiomyomas	Pelvic mass	Cervical endometriosis (histopathology)	Dienogest, antibiotics, and planned surgery	Not specified	[15]
2	Toniyan et al., 2023	Russia	46	Asymptomatic; regular menses	Not reported	Endometrial polyp	Cervical endometriosis	Not reported	Not specified	[16]
3	Pečovnik et al., 2022	Slovenia	32	Extracyclic/contact bleeding	Not reported	HSIL (CIN3)	Cervical endometriosis (histology)	Not reported	Not specified	[17]
4	Swetha et al., 2020	India	45	Intermenstrual bleeding	CT/MRI: possible malignancy	Gynecological cancer	Cervical endometriosis	Total abdominal hysterectomy + bilateral salpingo-oophorectomy	Not specified	[18]
5	Suba et al., 2019	Romania	24	Infertility, dysmenorrhea, and chronic pelvic pain	Not reported	Cervical dysplasia	Cervical and vaginal endometriosis (laparoscopy)	Laparoscopic excision	Not specified	[4]
6	Park et al., 2011	South Korea	54	Asymptomatic post-menopause	Not reported	Myoma uteri	Cervical endometriosis (histology)	LAVH	Benign, incidental finding	[19]
7	Kwek et al., 2010	Singapore	48	Postcoital bleeding	US: 4 cm cervical mass with vascularity	Cervical tumor	Polypoid cervical endometriosis	Surgical excision	Not specified	[20]
8	Yokota et al., 2008	Japan	37	Severe dysmenorrhea and regular cycles	Not reported	Adenocarcinoma (cytology)	Cervical endometriosis (biopsy)	Not reported	Not specified	[21]
9	Wang et al., 2024	China	42	Recurrent vaginal bleeding	MRI: cystic cervical lesion	Cyst/polyp	Endometriotic cyst of cervix (histopathology after excision)	Surgical excision + oral dienogest	8 months, symptom-free	[22]

## Data Availability

The datasets are private, but de-identified data may be provided upon request from the corresponding author.
